# Applying occupational and organizational psychology theory to entrustment decision-making about trainees in health care: a conceptual model

**DOI:** 10.1007/s40037-017-0336-2

**Published:** 2017-03-10

**Authors:** Ylva Holzhausen, Asja Maaz, Anna T. Cianciolo, Olle ten Cate, Harm Peters

**Affiliations:** 1 Dieter Scheffner Center for Medical Education and Educational Research, Charité-Universitätsmedizin Berlin, Berlin, Germany; 2 Department of Medical Education, Southern Illinois University School of Medicine, Springfield, IL USA; 3 Center for Research and Development of Education, University Medical Center Utrecht, Utrecht, The Netherlands

**Keywords:** Entrustment, Entrustable Professional Activities, Trust

## Abstract

In medical contexts around the world, supervising physicians continuously decide what degree of supervision to apply as trainees carry out professional activities. Although the implications for patients can be far-reaching, little is known about how these entrustment decisions are formed. The concept of ‘Entrustable Professional Activities’ has initiated interest and valuable research on factors that may influence the entrustment decision process.

The aim of the current article is to link models of entrustment developed in the fields of occupational and organizational psychology and military psychology to medical education studies that have explored the factors influencing physicians’ entrustment decisions. We provide a conceptual framework of the entrustment decision-making process, which we suggest will contribute to the understanding of how supervising physicians arrive at the decision to entrust a medical trainee with a professional activity.

## Introduction

Health care and health care training build on the progressive granting of responsibility and autonomy to learners, a practice with world-wide prevalence [1]. At certain points in their education, medical trainees are expected to have attained sufficient competence to carry out clinical activities unsupervised. An essential part of granting trainees progressive independence is the supervisor’s decision to entrust the trainee with specific activities. These daily decisions are also referred to as ad hoc entrustment decisions [2].

Such an ad hoc entrustment decision is often made implicitly, and is often guided by clinical service needs [2]. Take for example a physician supervising a senior medical student taking a history on a 29-year-old female patient with a cough. This supervisor must decide the degree of oversight needed to ensure the trainee gathers sufficient and accurate information to formulate a safe and effective diagnostic and treatment plan. Does the physician need to be present in the exam room to observe the encounter directly or even participate in the interview? Or is a higher level of trust and a lower level of supervision acceptable, such that the supervisor judges from the trainee’s post-encounter patient presentation that the student was able to do this well? What if the patient was a 49-year old-male patient also complaining of chest pain? How would the supervisor adjust his entrustment strategy then? These varying entrustment decisions are a matter of daily clinical routine, but are not well understood in detail.

Increasing interest in the concept of ‘Entrustable Professional Activities’ (EPAs) [3–5] has led to a heightened awareness of the supervisor-trainee entrustment decision process [6]. Ten Cate [7] describes EPAs as essential units of clinical work independently executable by qualified personnel. They require adequate knowledge, skills, and attitude, and they are, in their process and outcome, observable and measurable. EPAs can be entrusted to be performed by trainees under five different levels of decreasing supervision. The higher the level, the more independently the trainee is allowed to perform the EPA [8]. Ten Cate introduced the EPA concept in 2005 in response to concerns about the adverse effects of implementing competency-based assessment frameworks in medical education [9]. The competency-based assessment movement has resulted in the development and global implementation of competency frameworks such as that of the Accreditation Council for Graduate Medical Education [10] and the Canadian Medical Educational Directions for Specialists [11]. However, competency-based assessment of medical trainees has proven challenging [12, 13]. Critics point out that medical competence is more than the sum of separate competencies [14, 15]. EPAs have been introduced as a way to arrange, observe and assess medical competencies in a holistic manner. They integrate multiple competencies and, conversely, competencies map to multiple EPAs. A trainee who has mastered all EPAs of a specialty may generally be assumed to possess all relevant competencies of that specialty. More and more medical departments are now adopting ten Cate’s approach and have characterized sets of EPAs in their specialties, such as family medicine, internal medicine, paediatrics, psychiatry and geriatrics [16–21].

As entrustment decision-making is an essential part of the EPA concept, research has been initiated to investigate the process underlying the supervisor’s decision to entrust medical trainees with professional activities. Research conducted in medical education has mainly focused on identifying factors influencing the entrustment decision [6, 22–26]. Hauer et al. [27] conducted a study to investigate how entrustment decisions are shaped by these factors. They proposed a model of trust formation, which depicts the starting point and the outcome of trust, as well as accelerators and barriers to trust formation. Ten Cate et al. [2] provided an overview of different types of trust and entrustment decisions and factors involved in the entrustment decision process.

These valuable studies help to build understanding of entrustment decision-making, which represents a pervasive part of the clinical routine and the training of young physicians. What is not deeply understood is how the various factors influencing entrustment decision-making interact and how context influences their relevance and interplay. One way to build this understanding is to analyse individual supervisors’ stepwise thinking when considering the entrustment of a professional activity to a trainee in different contexts. This analysis should clarify how entrustment decisions are actually made and how different clinical contexts affect the size and importance of influential factors. It may also lead to the identification of additional influential factors. Ultimately, this understanding could be used to support more accurate, safer, and fairer entrustment decisions.

While research on trust in health professions education is relatively new, researchers in other domains have devoted substantial energy to studying entrustment processes. In this article, we introduce one leading trust model, developed by organizational psychologist Roger Mayer and his colleagues [28], as well as a modified version applied to understanding interpersonal interaction in a complex, high-stakes, interprofessional context: military command and control [29]. We combine these models with the findings of medical education studies of entrustment. Our aim is to expand understanding of the entrustment decision-making process in health care education to account for how ad hoc entrustment decisions are actually made and how context influences the decision-making process. We provide a research agenda to test and refine our explanations.

## Conceptualizing trust

**Fig. 1:**
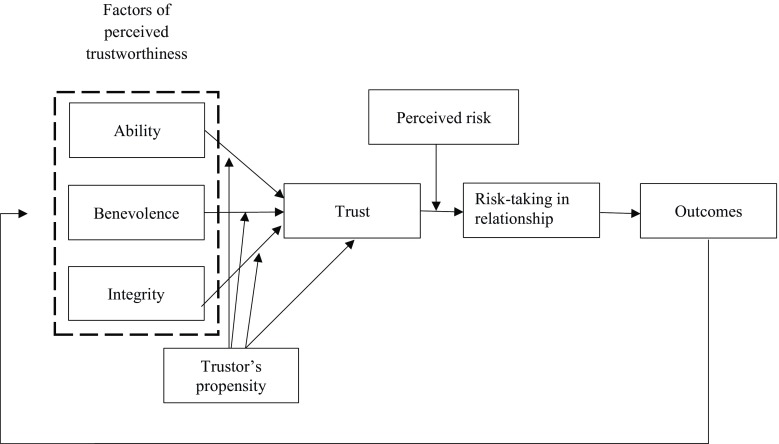
Model of Trust by Mayer, Davis and Schoorman [28]

In 1995, Mayer et al. [28] addressed heightened interest in understanding trust in occupational settings, which was triggered by increasing workforce diversity and the emergence of self-directed working teams. They developed a conceptual model of trust (Fig. 1), characterizing how it develops among two parties: a trusting party, called *trustor* and a party to be trusted, called *trustee*. This model was then applied to examining the development of employees’ trust in their managers, i. e. upward in hierarchy [30–32]. In comparison, in medical education the primary interest lies in how the supervising physician entrusts a trainee, i. e., downward in hierarchy. However, Mayer et al.’s model appears to be applicable to trust processes independently of hierarchy [32] and provides the opportunity to better understand the supervisor’s decision to entrust a trainee.

Mayer et al. [28] define trust as the ‘willingness of a party to be vulnerable to the actions of another party’ (p. 712) and hence as the ‘willingness to take risk’ (p. 712) in a relationship. A key characteristic of Mayer et al.’s model is the discrimination between factors promoting trust, trust itself, and outcomes of trust. The antecedents of trust proposed by Mayer et al. are characteristics of the trustor and the trustee. These include the perceived trustworthiness of the trustee, defined on the basis of his or her perceived *ability, benevolence* and *integrity* as well as the trustor’s propensity to trust, that is, his or her general willingness to be vulnerable to others. Propensity to trust is thought to be a stable characteristic akin to a personality trait. According to Mayer et al. [28], ability can be described as ‘skills, competencies, and characteristics that enable a party to have influence within some specific domain. The domain of the ability is specific because the trustee may be highly competent in some technical area, affording that person trust on tasks related to that area. However, the trustee may have little aptitude, training, or experience in another area (…).’ (p. 717). Benevolence and integrity are defined as more general dimensions of the relationship between trustor and trustee. ‘Benevolence is the extent to which a trustee is believed to want to do good to the trustor, aside from an egocentric profit motive. Benevolence suggests that the trustee has some specific attachment to the trustor.’ ([28], p. 718). Integrity is defined as ‘the trustor’s perception that the trustee adheres to a set of principles that the trustor finds acceptable.’ ([28], p. 719).

The trustor’s propensity to trust and the trustee’s perceived trustworthiness influence the trustor’s intent to be vulnerable to the trustee’s actions. The translation of intent to action, actual risk-taking, is influenced by the perceived risk of the trusting behaviour: ‘If the level of perceived risk is greater than the level of trust, the trustor will not engage in the risk-taking in relationship.’ ([28], p. 726). The outcome of this trusting behaviour in turn is expected to affect future trustworthiness judgments.

Mayer et al. [28] differentiated between the willingness to take risks (trust) and actual risk-taking (trust-related behaviour). They emphasized that willingness to take risks is attitudinal and could therefore be measured using questionnaires. In contrast, actual risk-taking can only be assessed via direct observation. This distinction is important, as a given behaviour might reflect factors other than the judgment of someone’s trustworthiness. For instance, an employee perceiving his manager to be untrustworthy may nevertheless act as trusting because the power differential leaves no other option. In health care, a supervisor may entrust an inexperienced trainee with an urgent task because no one else is around, or she may not trust an experienced trainee because of prior entrustment decisions with bad outcomes. Mayer et al. [32] argued for the general applicability of their model and discussed its extensive application to a wide range of contexts such as agribusiness, finance and political science. Yet, they have critically pointed out that it largely omits the impact of context on behaviour.

Cianciolo et al. [29] argued that previous trust research has failed to find a consistent relation between intent to trust and actual risk-taking behaviour due to disregard of context. When context is not clearly defined, situational characteristics can make trust-related behaviour difficult to recognize and seem unpredictable. In an attempt to develop behavioural measures of trust within military command and control organizations, Cianciolo et al. [29] extended Mayer et al.’s trust model by introducing the concept of ‘unit of analysis’ as a way to meaningfully differentiate contexts and reduce intervening variables between intent to trust and the degree of risk-taking. They adopted Mayer et al.’s definition of trust, but stressed that what entrustment ‘looks like’ behaviourally depends on the conditions under which it is enacted. For example, in tightly knit, homogenous teams executing well-defined tasks, high levels of trust may be inferred from economical communication among teammates because each can anticipate the other’s information needs. In diverse teams with complex, ill-defined tasks, high levels of trust may be inferred from fluent information sharing because participants identify as a team and share common goals. Health care examples of this phenomenon have been documented in Sutcliffe et al. [33], Pullon [34] and Lancaster et al. [35]. In both cases, prioritizing the group task at hand over managing interpersonal relations is a form of risk-taking, but willingness to be vulnerable appears different due to contextual factors.

In summary, the presented models of trust feature several characteristics that we believe are important for a research agenda that advances understanding of physician supervisors’ entrustment decision-making. They distinguish between trusting attitude on the one hand and actual, observable trust-related behaviour on the other hand. In these models, trust-related behaviour is the outcome of a context-bound entrustment decision-making process that is preceded by an accumulation of smaller decisions. These models propose a conceptual structure for the decision-making process which could be helpful for characterizing how and when supervisors’ intent to entrust results in the entrustment of a professional activity.

## Integrating trust theory with empirical findings on medical entrustment decisions

**Fig. 2:**
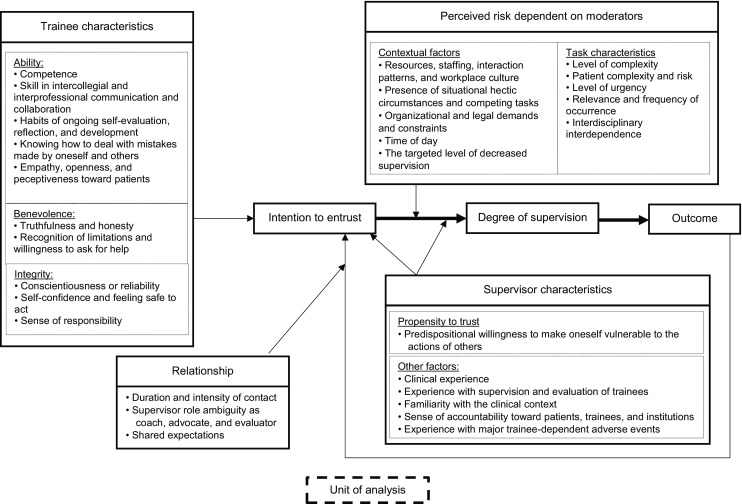
Conceptual framework of the entrustment decision-making process

Four categories of factors have been identified in a range of studies to influence entrustment decision-making: trainee characteristics, supervisor characteristics, characteristics of the task at hand and contextual factors [23–25, 36]. In addition, the relationship between the supervisor and the trainee has been identified as an important category [6, 27]. These studies provide a long list of factors in each category, summarized in an overview table by ten Cate et al. [2]. Fig. 2 illustrates how the findings of ten Cate et al. may be integrated with the trust models developed by Mayer et al. [28] and Cianciolo et al. [29] in a unified conceptual model of the entrustment decision-making process.

In this model, the intention to entrust a professional activity is influenced in part by characteristics of the trainee (trustee), which are summarized under Mayer et al.’s broad categories of ability, benevolence, and integrity. Characteristics of the supervisor (trustor) are also important. In their model, Mayer et al. focused on the trustor’s propensity to trust. This trait has also been found to affect medical entrustment decisions, but additional factors, such as the supervisor’s experiences with the trainee, have been shown to exert an impact [2]. Mayer et al. described how the relationship between the trustor and the trustee longitudinally influences the development of trust [28]. Ten Cate et al. [2] also assert that the duration and intensity of the supervisor-trainee relationship are important. The supervisor gains an impression of the capabilities and the personality of the trainee, which will influence future entrustment decisions. The longer the contact with the trainee, the better the supervisor can estimate whether the trainee has the capability to perform a professional activity. If the supervisor does not have much contact with the trainee, he or she might rely on credentials or the first impression of the trainee. On the development of trust, ten Cate et al. [2] distinguish between presumptive trust (based solely on credentials), initial trust (based on the first impression) and grounded trust (intensive contact with the trainee). In addition, supervisor role ambiguity as coach, advocate, and evaluator and the shared expectations between the supervisor and the trainee also seem to have implications for their trusting relationship.

The entrustment is posited to depend on the degree of perceived risk, which is influenced by situational circumstances and the nature of the professional activity [28, 29], as well as the supervisor’s characteristics [29]. Research on medical entrustment decision-making has identified contextual factors that may influence perceived risk, such as time of day and available assistance, and task characteristics, such as complexity and level of urgency. The degree of entrustment is expressed by a higher or lower level of supervision.

Cianciolo et al. [29] emphasized the importance of specifying how contextual factors influence the entrustment decision-making process. Clearly defining the context in which particular entrustment decisions are made strengthens the link between a supervisor’s judgment that a trainee is trustworthy and his or her degree of observed supervision. This enables more confident conclusions that a given level of supervision stems from the attending’s judgment vs. a unique and nonreplicable set of conditions. Cianciolo et al. [29] proposed the concept of ‘unit of analysis’ to refer to distinct ecologies whose contextual features differentially influence the factors affecting entrustment intentions and opportunities to mitigate risk when trustworthiness judgments are not 100%. In their work with military command and control teams, Cianciolo et al. identified three units of analysis that differed in their degree of interdependence among actors, geographical distribution between actors, and task complexity. In medicine, units of analysis, or trust ecologies, might differ along the same dimensions such that exerting supervisory control looks different and is less sensitive to trainee characteristics in intensive care units staffed by categorical residents over the night shift than in primary care clinics staffed by resident-attending teams during business hours. Units of analysis might also differ by specialty. Tiyyagura et al. [36] found, for example, that parental preferences influence supervisors’ decisions to entrust paediatrics trainees. Attempting to define units of analysis makes it possible to explore how exactly ‘context matters’ to the entrustment decision-making process.

Let us apply our framework to the EPA of ‘history taking’. The supervisor will first have to judge the trainee on his or her ability, benevolence and integrity. These judgments might depend on reputation or experience with the trainee. The supervisor has to decide whether the trainee is generally capable and trustworthy of taking a valid and conclusive history of a patient. Is the trainee capable of talking to the patient without the patient getting upset? Is there a chance that the trainee will confuse the patient? Could the trainee miss a significant finding? How reliable will the trainee’s report and differential diagnosis be, and is the supervisor willing to base future actions on it? Additionally, characteristics of the supervisor will influence the intention to entrust the trainee. Is he or she generally a trusting person? What experiences does he or she have with this trainee or with trainees generally?

Once the supervisor has formed the intention to entrust the trainee, it depends on factors related to the EPA and the context whether and to what extent the trainee will actually be entrusted. How difficult/complex is the patient? Has the trainee sufficient experience with this sort of case? How urgent is the situation? How long has the trainee been on the ward? The supervisor will have to weigh the risk of something going wrong against his belief that the trainee can manage this patient on his own. Dependent on all these judgments, the supervisor will estimate the degree of supervision required. The outcome of this decision will in turn influence future interactions with the trainee [2].

## Discussion

The aim of this article was to provide a conceptual framework to deepen our understanding of how supervising physicians arrive at ad hoc entrustment decisions in clinical practice. Following the recommendations made by Bordage [37], the use of frameworks is an attempt to identify potentially important variables and their interrelatedness and to make these assumptions explicit and testable.

In order to provide a conceptual basis for understanding how a clinical supervisor arrives at the decision to actually entrust a clinical trainee with a professional activity, we utilized factors identified through empirical research in medical education and combined these with theoretical models on trust from the fields of organizational and occupational psychology. From the current research we obtained a valuable overview of the factors influencing the supervisor’s entrustment decision. What we aimed to provide with our conceptual model is an understanding of how these factors might interact and under which circumstances the intention to entrust results in a particular level of supervision of the trainee. This information could be used to improve the accuracy and fairness of intentions to trust as well as the effectiveness of supervisory control.

One important task for future research is to explore which variables exert an influence on the entrustment decision and at which point. Mayer et al. [28] stress the importance of clearly defining and differentiating between factors contributing to trust and trust itself. Applying this argument to our proposed model, it also seems important to come to an agreement on definitions and labels for the variables involved in the entrustment decision-making process. We need to understand which factors mainly influence the *intention* to entrust a trainee and which ones influence the degree of *supervision provided to the trainee* (behaviour). We have provided an example of a possible chronological description of a supervisor’s thinking while entrusting a trainee with the EPA ‘history taking’. Research studying real-time chronological thinking observations would further add to the understanding of the entrustment decision.

A related area of research is identifying the units of analysis or trust ecologies that simplify the variables involved and allow confident inferences about trust intentions from observed supervisory behaviour. We propose department or specialty as a possible unit of analysis. This might be explored via ethnographic study followed by empirical studies that compare specialists’ entrustment decisions in response to different trust scenarios [38].

Additionally, it is not yet clear how strong the effects of various factors are. Teman et al. [26] asked attending surgeons to estimate the impact of various factors on their decision to trust a general surgery resident. This could be extended by manipulating trainee factors within an experimental study and testing the effect on physician’s willingness to trust. Field studies or studies conducted with simulated clinical situations within a ward could also be of high value.

Another research area is the identification of unknown influential variables in the entrustment decision-making process. The variables included in the model are those which have been considered as important in the entrustment decision-making process in the medical context [2]. The studies in medical education conducted so far have used either focus groups [25], Delphi studies [39], or questionnaires and interviews in combination with video-taped case vignettes [22, 23]. However, cognitive psychology research has pointed out that retrospective reports and general statements on cognitive processes have only limited validity as they might not reveal all influential variables [40, 41]. On the one hand, verbal reports might provoke reactive effects [40]. Asking a physician to describe how he judges whether the trainee in a case vignette is able to perform an EPA without supervision might focus his attention on factors which are easy to articulate and easily accessible. On the other hand, subconscious influential factors might not be detected [40]. Subconscious factors within the trustor are partly included in the trust models by Mayer et al. [28] and Cianciolo et al. [29]. Mayer et al. discussed the propensity to trust as an influential variable, and Cianciolo et al. name personality traits such as neuroticism or agreeableness as possibly having an impact. The influence of propensity to trust has been supported in medical entrustment research [2], and it is possible that additional subjective subconscious variables such as the first impression of a trainee or mood and gender of the supervisor have an impact [42]. Supervisors should be aware of potential subconscious variables and be able to differentiate between them. Intersubjectivity in supervisors is being acknowledged as valuable, because full objectivity can never be obtained [43]. The positive effect of gut feeling has been documented by studies [44], but supervisors should be in the position to identify potential biases and to correct for them [42].

Consequently researchers must be careful in choosing the method to study variables influencing the entrustment decision-making process. Research should apply those methods which yield most information about the unconscious and subjective factors.

The combination of trust theory with research on entrustment decision-making contributes to a model-based understanding of the entrustment process. Making entrustment decisions more transparent will eventually result in better grounded entrustment decisions and hence enhanced patient safety.

Cianciolo and Kegg [38] present a model of effective entrustment decision-making which focuses on understanding and improving supervisors’ risk-mitigation strategies. They propose that effective entrustment decisions could be reached by relying more on trainee observations and by accounting for subjective factors such as supervisors’ characteristics. However, this model remains theoretical, and the size of the effects of these variables on the entrustment decision-making is unknown. Our present model highlights the conceptual structure of the entrustment decision-making process and the influence and interaction of variables, yet further work is required to test the established propositions. Conceptual models are thought to be dynamic, and we have provided some suggestions for a research agenda which could challenge and alter the framework. We propose that this model will be helpful to clarify and deepen our understanding of the medical entrustment decision-making process.
